# Lacan on Trauma and Causality: A Psychoanalytic Critique of Post-Traumatic Stress/Growth

**DOI:** 10.1007/s10912-020-09622-w

**Published:** 2020-05-22

**Authors:** Colin Wright

**Affiliations:** Department of Cultural, Media & Visual Studies, University of Nottingham, B41a, Trent Building, University Park, Nottingham, NG7 2RD UK

**Keywords:** Lacan, Trauma, PTSD, Causality, Human rights

## Abstract

This article makes the case for the largely unacknowledged relevance of the thought of the French psychoanalyst, Jacques Lacan, for the emerging field of the medical and/or health humanities. From the 1930s all the way through to the late 1970s, Lacan was deeply concerned with the ethical and political consequences of then-dominant conceptions of the human in the ‘psy’ disciplines. His attempt to ‘humanise’ these disciplines involved an emphasis on humans as *symbolic* beings, inevitably entangled in the structures of speech and the ‘logic of the signifier.’ This article explores the implications of Lacan’s linguistic framework for his understanding of trauma. It argues that the Lacanian concept of trauma offers a timely antidote to dominant psychiatric notions of trauma today, linked as they are to the questionable politics of ‘Post-Traumatic Stress Disorder’ and, more recently, of ‘Post-Traumatic Growth.’

The term trauma has become something of a cultural lightning rod since the mid-twentieth century, attracting enormous attention in the fields of cultural and social theory as well as psychology and psychiatry. We have seen the rise of the discipline of Trauma Studies which touches on everything from the Holocaust to Rwanda, from the Truth and Reconciliation Commission in South Africa to post-9/11 (Caruth 2017; Casper and Wertheimer 2016). Regarding the psy disciplines (Rose 1998), we have seen early acknowledgements of traumatic memory, links between shell-shock and the so-called ‘war neuroses,’ and more recently the recognition of ‘Vietnam Syndrome’ and then ‘Post-Traumatic Stress Disorder’ (PTSD). Like addiction (Wright 2015), the scope of PTSD has expanded considerably in the latest edition of the *Diagnostic and Statistical Manual* which creates an entirely new class of ‘Trauma and Stressor-Related Disorders’ (American Psychiatric Association 2013). The DSM-V also implies the transmissibility of trauma beyond direct personal experience of it: one can now be traumatised by witnessing or even hearing of the traumas suffered by others.^1^ This has led to ‘pilots’ of remote drones being diagnosed with PTSD, despite being physically far from the front line in the new conditions of postmodern warfare. Linked to this contagion effect perhaps, there are now innumerable online screening tools and even apps enabling anyone to self-diagnose PTSD.^2^ Given the US military budget set aside for compensating affected soldiers, as well as equivalents in the private health insurance sector, there is a certain ‘market’ in trauma. Without at all denying that there is real human suffering behind what the term tries to capture—on the contrary, in fact—I want to describe this contemporary proliferation as ‘trauma talk.’

Trauma talk involves invoking the charged signifier ‘trauma’ in ways that have concrete consequences in the world precisely thanks to the absence of a consistent referent. Such trauma talk circulates fairly easily, not because of its conceptual rigour or correspondence to an empirical reality necessarily but because it catalyses sets of values and practices that I want to argue perform operations of individuation relevant to broader neoliberal agendas. Trauma is linked, after all, to the figure of the *victim*, to the victim’s rights in a compensation culture, and ultimately to the geopolitics of human rights. Within the positive psychology movement led by Martin Seligman, PTSD has also been inverted into Post Traumatic Growth (Seligman 2011), arguably a psychological equivalent of the neoliberal principle that ‘every crisis is an opportunity’ in disguise (Mirowski 2013). Nonetheless, it remains talk in a technical sense: one must learn the language of trauma, whether through the DSM’s vocabulary of ‘intrusion,’ ‘avoidance’ and ‘hyper-arousal’ or through an engagement with self-help literature with its language of ‘survivors’ and ‘recovery.’ In the field of critical social theory, there has been an ethical discourse around the notion of trauma ever since Adorno declared the impossibility of poetry after Auschwitz (1990). By contrast, in today’s more diffuse and medicalised ‘trauma talk,’ there is a loquaciousness that deserves to be characterised as a ‘discourse’ in Michel Foucault’s specific sense (1980). The discourse of trauma talk in fact abhors silence, insisting that trauma must be verbalised.

For better or for worse, behind the success of ‘trauma talk’ is undoubtedly a certain reading of Freud. For Trauma Studies as a cultural and social theory, Freud is still credited with recognising the link between trauma and repressed or screen memories and also with trauma’s resistance to psychic representation at the individual and social level (Fletcher 2013; Eekhoff 2019). Even the psychiatric framing of PTSD retains a clear indebtedness to Freudian ideas about repression and repetition compulsion. But as a psychoanalyst, I have learned to always ask *which* Freud? There are many after all. Which one does ‘trauma talk’ invoke? Speaking very generally, it seems to be the Freud of the very early Seduction Theory which can be dated back to a paper delivered in April 1896, entitled ‘The Aetiology of Hysteria’ (though two previous publications, ‘Heredity and the Aetiology of the Neuroses’ [1962a] and ‘Further Remarks on the Psych-Neuroses of Defence’ [1962b], had already sketched the argument). What Freud outlined in these so-called Seduction papers is indeed a post-traumatic model of neurosis, but one which, crucially, is not psychoanalytic.

Freud’s Seduction Theory arose from his early clinical work with hysterics in collaboration with Josef Breuer (Freud and Breuer 1991). Because free association seemed to reverse the repression of erotically charged material, Freud postulated an aetiology for hysteria based on a model of traumatic *memory*: these women, he believed, had suffered actual experiences of sexual abuse, but the resulting memory traces had been repressed from consciousness. Finding no psychic representation in the memory system, this traumatic imposition from the outside, of a sexuality for which these subjects were not developmentally ready, was both repressed and expressed in their conversion symptoms, as if their bodies spoke of what their minds could not confront. The classic example among Freud’s case studies is ‘Emma,’ a young woman who presented with a phobia of entering shops. During analysis, she was able to access a repressed memory from her eighth year when she was molested by a shopkeeper. Most of the elements of trauma as now popularly conceived are already present in this Freudian example:

1. a terrible shock ‘hits’ the organism from outside;
2. this shock is linked to either the threat of death or imposed sexuality;
3. it cannot be processed through existing frameworks of meaning;
4. yet the repressed returns in repetitive symptomatic forms (for Emma, her phobic panic attacks triggered by shop doorways).

As well as establishing these foundational elements, Freud lent impetus to the notion that the very act of talking about a trauma can potentially cure it. This fits well with today’s therapeutic culture which has commodified this aspect of Freud to the point that the company British Telecom could turn it into a marketing slogan in the 1980s—‘it’s good to talk.’ But what is forgotten here is the peculiar specificity of talking in analysis under conditions of transference and free association, very unlike everyday communication. Clinically speaking, it is certainly not the case that it is always ‘good to talk’ too quickly or too directly about a trauma. What is also forgotten is that Freud utterly renounced his Seduction Theory and had to, in order go on and invent psychoanalysis proper.

In a letter to Wilhelm Fliess dated the 21st of September 1897, Freud explicitly abandoned the Seduction Theory (1985). It had become apparent to him that the sexual content arising from free association did *not* always relate to repressed memories of real events. He was moving away from the restrictive model of experiential memory in order to better describe an unconscious he increasingly realised observed distinct laws. He therefore came to postulate that the sexual nature of these hysterical ‘memories’ related not to real experiences per se but instead to Oedipal fantasies and to the constitutively libidinal nature of the unconscious itself. For good reasons, this move has been a controversial one (Masson 1992), but only by abandoning the early Seduction Theory could Freud make the truly revolutionary arguments he developed in his *Three Essays on Sexuality* (1977). Whereas the pre-psychoanalytic Seduction Theory presented the trauma of sexuality as an external contingency, shocking the psychic system from without, the *libidinal* theory of the unconscious made the trauma of sexuality innate and primordial, giving proper primacy to what Freud called *psychic* reality. What today’s consumer culture veils, precisely in its commodification of sexuality as a lifestyle choice and a right to endless enjoyment, is the darker but more properly Freudian point: namely, that human sexuality *is* unavoidably traumatic.

Given that the DSM IV classified PTSD as an ‘anxiety disorder,’ it is worth pointing out that one can observe a similarly partial reading of Freud’s ideas on anxiety in some Cognitive Behavioral Therapy (CBT) frameworks. Rather than recognising the psychic reality of a fear without empirical locus, as Freud was careful to do in *Inhibitions, Symptoms and Anxiety* (1985), such CBT frameworks tend to reduce anxiety to a once realistic fear of empirical danger, the attendant signals of which have been misperceived. It was this understanding of anxiety as an irrational fear of fear that led to the notion of therapeutic ‘immersion’ or ‘flooding’ by means of which an experience that would correct the individual’s relation to reality could be induced. Whether in psychiatric conceptualisations of trauma per se or anxiety, one can discern the influence of behaviourism and a fairly naïve stimulus-response model, and very similar notions are now articulated through the alternative vocabulary of neuroscience and neuronal ‘plasticity’ (Abi-Rached and Rose 2010).

In these deviations from Freud, sometimes in the name of another Freud, I would argue that what is really at stake, ethically and eventually politically, is two quite incompatible models of *causality*.

## Traumatic causality

In common with Freud’s pre-psychoanalytic Seduction Theory, PTSD conceptualises trauma in terms of a fairly linear, Newtonian—or Pavlovian—causality. In the vocabulary of the DSM, there is an initial ‘stressor event’ which *then* goes on to have pathological consequences such as persistent re-experiencing in nightmares, hyper-arousal and extreme avoidance behaviours. A, that is, leads to B. Because of its historical and institutional links to war, there is much less scope today for the idea that the stressor event could be unconscious. The victim of trauma generally knows what the traumatic event was, and the almost juridical emphasis is on demonstrating the causal link between it and subsequent disordered behaviour. There is something paradoxical, therefore, about ‘trauma talk.’ On the one hand, it foregrounds the horror of the unexpected, the unpredictable, the out-of-the-blue. Yet on the other, it posits a cause-and-effect relation that is determinate enough to make of the traumatised individual a helpless victim of unfortunate circumstances. The circularity, as it were, of this linearity, can clearly be seen in its reversal: a great deal of research now goes into identifying genetic risk factors that *pre*dispose for PTSD.^3^ Psychometric as well as physical tests for the military attempt to screen for such predispositions, and ‘psychological briefings’ have been used to prevent the onset of PTSD immediately after conflict. This push for early intervention and preventative medicine fits well both with neoliberal cost-benefit analyses of healthcare provision (Polzer and Power 2016) and with what Ulrick Beck has called ‘risk society’ (2009). Not only armies but governments now present themselves as capable of eradicating contingency within administrative frameworks of predictability.

However, staying closer to Freud’s properly psychoanalytic abandonment of the Seduction Theory yields a profoundly different model of causality. Freud himself claimed to be constitutively incapable of doing philosophy. While the proximity of some of his ideas to those of Schopenhauer and Nietzsche permit us to doubt him on this score, it was nevertheless his fidelity to his clinical practice, rather than a taste for abstraction, that led him to sketch a non- or anti-Pavlovian theory of psychic causality. The transitional text, *Beyond the Pleasure Principle*, of 1920 emerged from clinical work with victims of shell shock after WWI. In it, he both revised the pleasure principle in the face of the repetition compulsion and posited the controversial death instinct. But as usual with Freud’s work, one can find the seeds of these later elaborations even in the earliest writings.

Let us take the case-study of ‘Emma’ again. Already, Freud had recognised a recursive relationship between the two elements that Emma, after analysis, was able to bring in to relation: intuitively, we would place the inappropriate touching she, at the age of eight, experienced at the hands of the shopkeeper as the ‘stressor event’ that then led to a pathological manifestation five years later at age thirteen when she felt immense anxiety on perceiving that two shopkeepers laughed at her for no discernible reason. A seemingly leads to B, then. But Freud focuses on the gap of five years between these two events and thus on what he terms the *nachträglich*, or ‘belated,’ nature of Emma’s trauma. If linear causality is retained, it is only by being split in two. The shock of the ‘first’ event is registered in the psychic apparatus but only as a spike of uncathected libido, awaiting its subsequent symbolic cathexis. However, with this splitting of linear causality, Freud had already intuited that Emma’s trauma only came into being *retrospectively* when a link was made to the ‘second’ experience at age thirteen via the common trait of the leering smile: her phobia was thus a response to what registered as traumatic only *après-coup*. In other words, B leads to or gives rise to A.

To understand fully this aspect of Freud, it is necessary to turn to a much more philosophically inclined psychoanalyst, Jacques Lacan. More than any other thinker in the twentieth century, Lacan understood trauma as a ‘textuality,’ thus as adhering to what he called the ‘logic of the signifier.’ Whilst this logic separates word from thing, opening up a hole in the speaking being’s world and suspending naïve notions of ‘reality’ and its causal laws, it also makes this structural trauma amenable to symbolic treatment. Lacan develops this position at various points in his work.

In a 1946 paper entitled, ‘Presentation on Psychic Causality’ (2006a), Lacan vehemently attacks Henri Ey’s alleged organic determinism, appealing instead not just to psychoanalysis but to philosophy in order to make a distinction between, precisely, reality and truth. For human subjects, he argued, psychic causality is not a matter of the quantifiable reality physics has been able to model so impressively but rather, of a subjective *truth* that language can never fully capture. In a 1945 publication, entitled ‘Logical Time and the Assertion of Anticipated Certainty’ (2006b), Lacan drew amazingly early on game theory in order to bring out the recursive causality already implicit in Freud’s notion of *nachträglichkeit*. Using a complex thought experiment that there is not space to go into here (see Wright 2018 for an account of it), Lacan showed that even rule-bound, logical puzzles can only really be resolved by subjects who act, not because they subordinate themselves to the rules of the game, but because, in and through acting, they retrospectively create the certainty that allows them to leap into the unknown. In his second seminar of 1954 and 1955, he returned to the problem of causality, this time making use of cybernetics and information theory (1991). His fifteenth seminar would be entirely devoted to the psychoanalytic act, contrasting the conditioned causality of Pavlov’s dogs with Caesar’s crossing of the Rubicon (1982). However, I will concentrate here on what Lacan says about causality in his eleventh seminar, *The Four Fundamental Concepts of Psychoanalysis* (1994), held between 1963 and 1964.

In that seminar, Lacan undertakes a revision of the theory of causes outlined in Aristotle’s *Metaphysics*, where a distinction is made between two types of causality, *automaton* and *tuché*. These two terms have generally been translated—erroneously, according to Lacan—as fortune and chance respectively. ‘Automaton’ refers to a kind of machinic unfolding familiar today in the idea of a programme that runs, implicitly in a kind of loop and produces predictable results. Classically, we might conceive of automaton as fate which various Greek tragedies showed cannot be avoided. ‘Tuché,’ however, corresponds to a totally unpredictable encounter with what Lacan calls ‘the real,’ a concept which must not be confused with reality (for the same reason that Freud was so careful to specify a *psychic* reality). If experienced reality is reassuringly meaningful, predictable, automatic even, the real is incompatible with any such symbolic co-ordinates: it cannot be predicted or represented or spoken fully, even after it has burst forth. This is why Lacan will later invent the neologism, ‘troumatisme,’ with ‘trou’ meaning hole. An irruption of the real in the midst of what had been our reality, trauma tears a hole in the very fabric of meaning. As horrifying as this can be, tuché can nonetheless be an experience with transformative implications that the subject can assume as her own. Fate can be replaced with a destiny that is in some sense subjectively chosen, as figured by Caesar’s crossing of the Rubicon. Tuché is not raw chance then, but an event, an always missed encounter, in the wake of which, the unconscious has the opportunity to construct something new.

Lacan illustrates this distinction through a reading of one of the dreams Freud reports in *The Interpretation of Dreams* (1991). A father suffers at the level of reality an experience which by most standards would count as traumatic: the death of his son. Exhausted, he asks an elderly friend to sit with his son’s body so that he, the father, can get some sleep. He then dreams that his son comes to him and demands, ‘Father, can’t you see I’m burning?’ upon which horrifying image, he awakes. Noises from the room next door alert him to the fact that a candle has, in reality, tipped over and caused a fire, of which the elderly man, who has himself fallen asleep, is completely oblivious. Where should we locate causality here? The common sense model places the cause of the father’s waking in the noises coming from the adjacent room, from reality, where accidents can happen but at least observe the laws of physics. Yet Lacan reverses this assumed relation by insisting that the cause of the father’s waking is to be found in that dreadful phrase, ‘Father, can’t you see I’m burning?’ This question is an accusation, a ‘why hast thou forsaken me?’, which speaks to the impossibility of predicting the real, even for a father who would love to be able to control everything in order to guarantee his son’s safety. “Is there not more reality”, Lacan asks, “in this message than in the noise by which the father also identifies the strange reality of what is happening in the room next door?”(1994, 58). The father’s own interpretation, that it must have been the actual fire that awoke him, is a kind of defensive appeal to reality in order to evade this glimpse of the real which piques his unbearable guilt and remorse. Indeed, in dealing with the actual fire, he gets to make amends, though in a more or less useless way, for what he hadn’t been able to do: to rescue his son from the fever that caused him to ‘burn up’ and die. Of the phrase in the dream then, Lacan says, “This sentence is itself a firebrand—of itself it brings fire where it falls—and one cannot see what is burning, for the flames blind us to the fact that the fire bears […] on the real” (59).

## Trauma and the human rights industry

There is always the danger of getting consumed and indeed burned by Lacan’s rather beautiful and yet often dazzling rhetoric, so I will close by bringing out what I believe to be at stake in the psychic causality he is dramatising here. I will do so, firstly, by referring very briefly to Ethan Watters’ excellent book, *Crazy Like Us: The Globalization of the Western Mind* (2011), specifically his account of PTSD counsellors in post-tsunami Sri Lanka. I will then outline some of the broader acknowledgements of the potential reductiveness of Western psychiatric nosology coming from the fields of critical and cross-cultural psychiatry. Finally, I will conclude by isolating what a Lacanian approach can usefully add to these existing critiques in order to outline Lacan’s potential contribution to the project of the medical humanities.

In the second chapter of Watters’ book, he describes a veritable army of trauma counsellors and researchers who raced to the scene of devastation within days of the tsunami striking Sri Lanka in 2010, convinced that a ‘second tsunami’ of PTSD was inevitably going to hit the country in the coming weeks. He argues that the image of this ‘second tsunami’ was sufficiently potent to mobilise an enormous therapeutic apparatus. So-called traumatologists speculated wildly in the international media, predicting that between 50 and 90% of the affected population would develop symptoms of PTSD and that up to 30% of those would be at risk of suicide (Watters 2011). Thousands of what one Sri Lankan called ‘parachute researchers’ were soon on the ground, conducting interviews and surveys, organising psychological debriefings and encouraging children to symbolise their distress through drawing and play. Some even randomly handed out anti-depressants as if the packets of pills were on a par with food aid packages.

Exposing the extremely problematic imbrication of clinical practice with the simultaneous production of a self-justificatory evidence base characteristic of much Western medicine (though perhaps CBT pre-eminently), Watters persuasively argues that this emergency ‘therapy’ was inseparable from the production of data. For example, ‘research’ on the extent of PTSD prevalence in Sri Lanka was conducted even before the time period—clearly specified in the DSM-IV’s diagnostic criteria—of at least one month of persistent symptoms. In this sense, the long juridical tradition of ‘emergency powers’ that suspend civil law in times of war or natural disaster was extended to the psychiatric laws codified in the DSM itself. The enormous humanitarian enterprise that was thereby able to unfold, clearly motivated by genuinely good intentions, was also driven by an urgent belief that human responses to trauma are universal and therefore homogeneous. The organism, whether in Sri Lanka or Seattle, was supposed to respond to traumatic stimuli in fundamentally the same way. Directly related to this confidence in an established and universal cause-effect relation, these volunteers were also imbued with a faith in Western medicine’s capacity to identify, intervene, and alleviate a standard checklist of PTSD symptoms. And yet, many trauma experts and researchers in Sri Lanka were soon confused to find that, though clearly shocked, bewildered and distraught, many victims of the tsunami did not conform to conventional PTSD criteria. Their interpretation of this finding? These people were clearly in denial.

Watters rightly points out the view of PTSD that these well-intentioned counsellors and researchers brought with them steamrollered the particularities of the Sri Lankan cultural context. I would argue this has a great deal to do with its implicitly Pavlovian causality in turn buttressed by behaviourist assumptions which nourish a particular view of what subjects are and how they should be governed. Yet Watters also notes that Sri Lankan culture, partly because of its long civil war, already includes considerable resources for dealing with trauma, from Hindu and Buddhist religious traditions to an emphasis not on the individual ‘resilience,’ ‘flourishing’ or ‘growth’ now favoured by positive psychologists but on group support and community relations. The very idea, then, of what a subject is and how he responds to distress, was fundamentally different.

In making these points, Watters is drawing on critiques of mainstream western psychiatric nosology that have already been mounted within comparative cultural psychiatry, often based on medical ethnography and anthropology. Shortly after the DSM first included PTSD in 1980 for example, Allan Young was already conducting ethnographic work with Vietnam veterans in the US which, he argued, showed that both ‘traumatic memory’ and PTSD were essentially discursive constructs enabled by institutional powers and practices, rather than transhistorical noslogical entities (Young 1997). Derek Summerfield has also argued that PTSD had a specific sociological function in the post-Vietnam US context, primarily that of re-presenting soldiers not as ‘baby killers’ who should be blamed for the atrocities of the war but as victims of a warmongering US government and therefore deserving of help and support (Summerfield 2001). The instrumental political utility of this category, seemingly present from its origins, has only become more transparent as it has become globalised. For precisely as neoliberal models of globalisation spread in the 1990s and a corresponding doctrine of supra-national human rights was given institutional form, PTSD started to be integrated into ‘human rights’ interventions in post-conflict situations outside the ‘west.’ Well before the Sri Lankan disaster that Watters details, the United Nations and the World Health Organisation had already jointly advocated the training and deployment of hundreds of ‘trauma advisors’ in the former Yugoslavia, and UNICEF launched a similar programme in post-genocide Rwanda aimed at traumatised children (UNICEF 1996). What I have called the ‘Pavlovian’ causality of PTSD then, as conceptualised and implemented in post-conflict relief programmes from the 1990s onwards, perhaps reduces suffering but certainly  does reduce agential subjects to the status of more or less passive victims. Such victims can then be said to be in need of better government than their ‘failed states’ could apparently provide. Trauma talk can thereby play its part in neoliberal forms of governmentality.

More attuned to cultural differences and cross-cultural miscommunication than the makers of the explicitly globalising DSM, critical psychiatrists have tried to contest the homogenising effects of the biological model of PTSD, both by pointing out the cultural specificity of ‘presenting problems’ in diverse locales and by formulating alternative descriptors which incorporate the socio-cultural dimension such as Kleinman’s notion of ‘social suffering’ (1997) and Eisenbruch’s ‘cultural bereavement’ (1991). These are important interventions that go a long way towards reintroducing not just social and cultural factors but crucially *political* contexts into the notion of traumatic causality, which the narrowly biological framework tends to radically downplay. However, there are also some limitations with this kind of work which, I would suggest, can be productively negotiated by entering into dialogue with the Lacanian approach. Firstly, cultural psychiatry runs the risk of sliding into a cultural relativism that has the potential to leave traumatised subjects alone with the resources supposedly available to them, even when research has shown that post-traumatic anxiety in non-western contexts often centres on very practical, material concerns of daily living which cannot be addressed by cultural and communal frameworks alone (Wilson and Drozdeck 2004). Secondly, cultural relativism, sometimes unconsciously tinged with exoticism, can also conflate individual experience with much broader anthropological assumptions, as if individuals were always prototypes of their cultures: clearly, this gesture is not innocent of the very universalising zeal that motivates the DSM (rather as ‘glocalisation’ is hardly incompatible with globalisation as such). Thirdly, whilst the importance of the cultural dimension is rightly stressed over and above a putative biological universality, underlying assumptions about causality often remain intact: the stimulus-response model, for example, can be retained alongside an acknowledgement of the cultural diversity of responses. At its worst, the ethical and political question of causality I have focussed on here is sidestepped by recourse to a crude ‘Foucaultian’ social constructionism that effectively replaces the notion of a traumatogenic stimulus or cause with a knowledge/power nexus within institutionalised medicine.

Lacan’s emphasis on the role of language and the impact of the logic of the signifier on psychic causality both underlines and enhances critical and cultural psychiatry. After all, Lacan’s emphasis on the symbolic dimension enabled him to avoid relativism (language is fundamental to the human condition *tout court* for Lacan) and also the more facile aspects of constructionism (if discourses are pushed to produce constructions, it is in response to something real). Thus, as early as the 1950s, Lacan was drawing on the structuralist anthropologist Claude Lévi-Strauss to argue that cultures are symbolic systems that effectively treat, through myths and rituals, a fundamental contradiction or impossibility that he himself would go on to call the ‘real’ (Lévi-Strauss 1969). Just as Lacan argues that subjects are already responses to the trauma of sexuality and castration in language then, so he agrees with Lévi-Strauss that cultures are similarly unique responses to a traumatic kernel and should be honoured as means of treating the real. From a Lacanian perspective, with speaking beings shaped by their cultural contexts, one is always dealing with a symbolic, and therefore recursive, causality that allows suffering individuals to transcend the status of victim of an unfortunate reality, to become subjects of their own speech. But to discern that singularity, one has to listen, not talk. This Lacanian perspective has a great deal to offer to the medical and/or heath humanities, which likewise aims to honour the testimonies of patients as a form of experiential evidence that, if attended to, can humanise some of the clinical and conceptual frameworks associated with ‘objective’ disease models of illness.

### Endnotes

^1^For a journalistic account of this see http://www.nytimes.com/2013/02/23/us/drone-pilots-found-to-get-stress-disorders-much-as-those-in-combat-do.html?_r=0. Accessed 18th January 2019.

^2^For just one example, see http://www.healthyplace.com/psychological-tests/ptsd-test/. Accessed 19th January 2019.

^3^See http://www.redorbit.com/news/health/1113311445/two-genes-linked-to-predisposition-for-ptsd-010915/. Accessed 19th January 2019.
